# Acceptability of Mebendazole Chewable Tablet in Children Aged 2 to 4 Years in Peru

**DOI:** 10.3390/pharmaceutics14010027

**Published:** 2021-12-23

**Authors:** Fernando Perez, Thibault Vallet, Zarela Bravo, Kristin Callahan, Fabrice Ruiz

**Affiliations:** 1 INMED Partnerships for Children/INMED Andes, 21630 Ridgetop Circle, Sterling, VA 20166, USA; fperez@inmed.org (F.P.); zbravo@inmed.org (Z.B.); kcallahan@inmed.org (K.C.); 2 ClinSearch, 110 Avenue Pierre Brossolette, 92240 Malakoff, France; thibault.vallet@clinsearch.net

**Keywords:** acceptability, medicine, chewable, formulation, pediatric, children, mebendazole, soiltransmitted-helminthiasis, deworming, ClinSearch Acceptability Score Test (CAST)

## Abstract

Soil-transmitted helminthiasis (STH) is among the most common of parasitic infections, affecting vulnerable populations in tropical/subtropical areas globally. In endemic countries, children, a high-risk population, require treatment and preventive interventions. Mebendazole, a WHO-recommended medicine, originally formulated as a tablet that was often crushed for administration to young children unable to swallow it, was reformulated as a chewable tablet. Acceptability is a key aspect for treatment effectiveness in pediatrics. Herein, we used a validated data-driven approach to investigate the acceptability of the 500-mg mebendazole chewable tablet in children aged 2 to 4 years in Peru. Observer-reported outcomes were collected for 182 medicine intakes. Acceptability was scored using the acceptability reference framework: a three-dimensional map juxtaposing “positively accepted” and “negatively accepted” profiles. Results found that the 500-mg mebendazole chewable tablet was classified as “positively accepted” in children aged 2 to 4 years. Acceptability increased with age and some acceptability issue remain for the younger children. Nevertheless, this formulation was considerably better accepted than the conventional tablets regardless of treatment in young children. This chewable formulation appears to be an appropriate alternative to the hard tablet of mebendazole for treatment of STH and preventive interventions in children aged 2 to 4 years.

## 1. Introduction

Soil-transmitted helminthiasis (STH) is the most prevalent parasitic infection among the neglected tropical diseases (NTD), with chronic impacts on health, social, and economic development of affected populations [1,2,3]. STH often goes unnoticed, but the disease constitutes a major public health problem, causing effects that are often difficult to quantify due to its contribution to other diseases such as anemia and malnutrition, and its high rate of reinfection. Nutritional deficiency produced by STH negatively impacts physical growth and development of infected individuals primarily due to loss of iron and protein, poor nutrient absorption (due to competition for vitamin A), loss of appetite and, consequently, deterioration of the physical condition [4].

A total of 24% of the world’s population, or approximately 1.5 billion people, are infected by soil-transmitted helminths. Some 267 million preschoolers and more than 568 million school-age children live in areas with high infection rates, requiring treatment and preventive interventions [4]. The populations most at risk for STH impacts are preschool and school-age children and women of childbearing age, as these populations are in a period of growth with high demand for nutrients.

Mebendazole and albendazole are the two World Health Organization (WHO)-recommended treatment drugs [4]. Although albendazole is available as a tablet or an oral suspension, mebendazole was originally formulated only as a tablet which was often manipulated (e.g., crushed) for administration to younger children who were unable to swallow it in solid oral dosage form (SODF). The WHO’s call for a more child-friendly formulation of mebendazole resulted in the development of a 500-mg chewable tablet (Vermox™) [5]. Safety and effectiveness of this medicine against *A. lumbricoides* (roundworm) and *T. trichiura* (whipworm) was established in children aged 1 to 16 years [6,7], as well as against hookworm in children aged 3 to 12 years [8]. Only the latter study explored the acceptability of both the new and the original mebendazole formulations. In pediatrics, medicine acceptability has emerged as a key factor of adherence and consequently, treatment effectiveness. The European Medicines Agency (EMA) has defined patient acceptability as, “the overall ability and willingness of the patient to use and its care giver to administer the medicine as intended” [9]. This multi-faceted concept is likely to be driven by the characteristics of both users (e.g., age, health status, culture) and products (e.g., palatability, swallowability, usability). While swallowability is crucial for conventional tablet, palatability (the overall appreciation of the medicine in relation to its smell, taste, aftertaste and mouth feel) is a key aspect for chewable tablet [10].

In the Americas, Peru is among the countries with the greatest prevalence of helminthiasis. Since 2017, Peru’s National Ministry of Health has provided mass drug administration (MDA) of antiparasitic (deworming) medicine to all people 2 years of age and older as a public health measure to control intestinal parasitic infection. In this context, a study on acceptability of 500-mg mebendazole chewable tablet in children was conducted by INMED Andes in 2019. Unpublished results showed that the acceptability of the chewable tablet by children increased with age, although it appeared to not be related to sex, population type (urban or rural), or ethnic group. Children from 5 to 11 years of age were found to sufficiently accept the chewable tablet. These results were in line with the study of Palmeirim et al. [8]. An important limitation of this study was the very limited number of children aged 3 to 5 years. Herein we used a validated data-driven approach—the ClinSearch Acceptability Score Test (CAST) [11,12,13,14,15,16,17,18]—to further investigate the acceptability of 500-mg mebendazole chewable tablet in the younger children aged 2 to 4 years.

## 2. Materials and Methods

### 2.1. Study Design, Objective, and Setting

This multicentric, cross-sectional, and observational study was conducted in Peru between May and June 2021. The objective was to generate evidence on the acceptability of the 500-mg mebendazole chewable tablet (Vermox™) among young children aged 2 to 4 years, and investigate the potential influence of age, sex, and population type (urban or rural).

Subjects were enrolled in selected sample preschools in urban (Yarinacocha) and rural (Manantay) districts of the jungle region of Ucayali, where STH infection is endemic, during Peru’s first national deworming campaign of 2021.

According to local regulations, the present study was approved by the Ethics Committee of the Ucayali Health Ministry on 21 May 2021 (FUT 0082452).

### 2.2. Participants and Sample Size

Subjects aged 2 years (≥24 to <36 months), 3 years (≥36 to <48 months), and 4 years (≥48 to <60 months) were treated for STH with the 500-mg mebendazole chewable tablet during the deworming campaign, with a written informed consent obtained from the parent(s)/legal representative. Children were not included in the study if they met one of the following exclusion criteria:

- Receiving or received other anthelmintic treatment in the last 6 months;
- Receiving metronidazole for any other diagnosed illness in the last 15 days;
- Known allergies to the compounds in 500-mg mebendazole;
- Presence or history of important systemic or chronic diseases, diagnosed by a doctor;
- Fever at the time of observation;
- Acute respiratory disease;
- Acute or chronic diarrheal disease;
- Children, parents, or people who live within the child’s household currently diagnosed with COVID-19;
- Congenital malformation of the mouth or palate that hinders oral administration of medication;
- Participating in another clinical or similar study concurrently.

A minimum of 30 evaluations of medicine intake were necessary to get an acceptability score with a satisfactory precision using the CAST methodology. Samples of 60 subjects (30 males and 30 females; 30 urban and 30 rural) per age group (2, 3 and 4 years) were targeted for participation.

### 2.3. Data Collection

Health personnel responsible for drug administration during the deworming campaign and specially trained for the acceptability study administered the chewable formulation to children in their homes or local facilities (due to COVID-19 related school closures). The tablets remained in their original packaging until they were administered to the children. Health personnel recorded the following objective measures on field data forms, including events/behaviors that were observed during the intake of the medicine under investigation:

- Results of intake (the required dose was fully, partly, or not taken);
- Patient reaction during the administration using a 3-point facial hedonic scale (positive, neutral, or negative reaction);
- Time needed to:
  o Prepare the dose (from opening the packaging to having a required dose of medication ready to use, including all handling and modifications),
  o Administer the required dose of medication (from a required dose of medication ready to use to the end of the intake);
- Dividing the intake of a prescribed dose of the medication which cannot be taken as a whole (e.g., several sips of an oral preparations);
- Altering the use, such as
  o Modifying the dosage form (e.g., crushing, dissolving a tablet),
  o Using another route/mode of administration;
- Using food/drink
  o The prescribed dose of the medication had to be mixed with unintended drink or food (e.g., gelatin, yogurt),
  o The child had to take drink or food just before or after the dose administration (e.g., eat a cookie to mask the drug taste, take a spoon of honey for easier swallowing);
- Using a device not provided with the medication (e.g., disposable spoon or cup);
- Promising a reward;
- Using restraint (i.e., the child was forced to take it).

For each method used to help with taking the medicine (the last six bullet points) the observers were requested to specify any further information in a text field (e.g., if the child used drink or food, what type of drink or food and the quantity). 

In addition, health personnel reported patient characteristics (sex, age, comorbidities and previous exposure to study treatment), context of use (person in charge of preparing and administering the medicine, place and time of administration), and any remarks on medicine use. 

The field data were entered into the electronic database system by the health personnel coordinators. Thereafter, data were reviewed by the data manager and data collection lead group. The principal investigator clarified any discrepancies with health personnel and their coordinators.

### 2.4. Data Analysis

#### 2.4.1. Data Table

In the data table, each row corresponded to one evaluation of the intake of 500-mg mebendazole chewable tablet by a specific patient, and each column represented one of the nine aforementioned observational variables (e.g., result of the intake) describing the many aspects of acceptability, with an observed measure (e.g., fully taken) being entered into each corresponding cell. The sum of preparation and administration times was transformed into a categorical variable and classified as short (1 min and less), medium (from 1 to 2 min and 30 s), or long (longer than 2 min and 30 s). In addition to the nine active variables included in the multivariate analysis, each row was associated with supplementary variables describing the patient and the context of use.

#### 2.4.2. Acceptability Reference Framework

In this study we used the acceptability reference framework: a three-dimensional map (3D-map) juxtaposing two distinct acceptability profiles—"Positively accepted” and “Negatively accepted”—materialized by green and red areas on the 3D-map, respectively. This intelligible tool was developed based on 1778 standardized evaluations, without missing observed measure, of the intake of multiple oral/buccal medicines in children under 12 years of age. These evaluations were previously collected in eight countries (France, United Kingdom, Germany, Norway, Poland, Morocco, India, and Japan) using the standardized questionnaire. As previously described [11,12,13,14,15,16,17,18], multivariate analysis mined this large set of evaluations: first, a multiple correspondence analysis (MCA) summarized the key information into the low-dimensional Euclidean space where proximities between elements express similarities; subsequently, hierarchical clustering on principal components and k-means consolidation gathered the most similar evaluations (the closest on the 3D-map) into two coherent and meaningful clusters defining the acceptability profiles.

The evaluations collected in this study were not included in the dataset of 1778 standardized evaluations that gave rise to the acceptability reference framework. They were included in the multivariate analyses as supplementary information, with no influence on the factorial method. Thus, the evaluations of 500-mg mebendazole chewable tablet from this study were plotted on the 3D-map, allowing for scoring process implementation.

#### 2.4.3. Acceptability Scoring

The 500-mg mebendazole chewable tablet was positioned on the reference framework at the barycenter of all its evaluations collected in this study and plotted on the 3D-map. Confidence ellipses surrounding the barycenter for all dimension pairs defined an area containing its true position with 90% probability if the experiment was to be repeated. If the barycenter, along with the entire confidence ellipsis surrounding it, belonged to the green area of the map, the medicine was classified as positively accepted.

No evaluation of the intake of the mebendazole hard tablet–original form–in children was collected during this study. The acceptability score of 500-mg mebendazole chewable tablet was thus compared to the average score of conventional hard tablets regardless of treatment in the age group of interest—children aged 2 to 4 years—to obtain a relative acceptability evaluation. This acceptability score was determined relative to previous evaluations from the dataset that gave rise to the acceptability reference framework. If confidence ellipses around distinct barycenters did not overlap on the map, acceptability scores were significantly different.

Subsequently, the evaluations of 500-mg mebendazole chewable tablet were partitioned into three subgroups according to the age of participating children (2, 3, and 4 years). Similarly, the influence of sex and population type (rural and urban) was investigated.

Pearson’s chi-squared test and Fisher’s exact test were used to determine whether there were statistically significant differences for the observational variables as well as the supplementary variables between the subgroups of children being compared.

Data analyses were performed using R version 1.0.136^©^ (RStudio Team (2016). RStudio: Integrated Development for R. RStudio, Inc., Boston, MA, USA). The R package FactoMineR [19] was used to perform mapping and clustering processes.

## 3. Results

### 3.1. Subjects

In this study, 182 evaluations of the intake of 500-mg mebendazole chewable tablet in children aged 2 to 4 years were documented. Table 1 presents the characteristics of the patients, stratified by patient age. As we might expect, there were significantly more children aged 2 years compared to the 3- and 4-year old who had taken the medicine for the first time. There was also a significant difference in term of time of administration, as more children aged 4 years had taken the medicine at mid-afternoon.

### 3.2. Overall Acceptability of 500-mg Mebendazole Chewable Tablet

**Figure 1:**
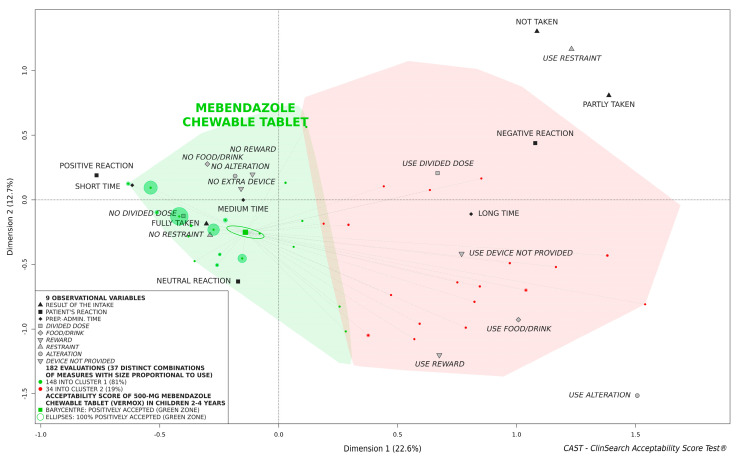
Acceptability of 500-mg mebendazole chewable tablet in children aged 2 to 4 years.

According to the acceptability reference framework, the 500-mg mebendazole chewable tablet was classified as positively accepted in children aged 2 to 4 years considered as a whole (Figure 1). Although some evaluations were positioned in the negative area, the barycenter of the 182 evaluations, along with the entire confidence ellipses surrounding it, was fully located in the green area of the 3D-map.

**Figure 2:**
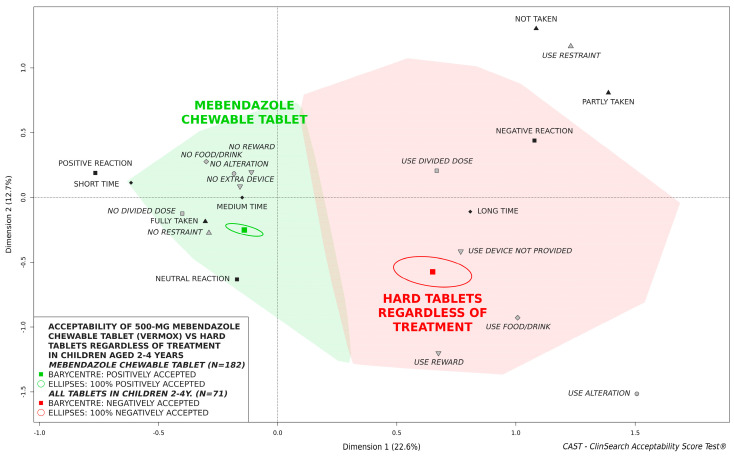
Acceptability of 500-mg mebendazole chewable tablet compared with tablets regardless of treatment in children aged 2 to 4 years.

Figure 2 shows the significant difference between the overall score of 500-mg mebendazole chewable tablet in children aged 2 to 4 years and the score of conventional hard tablets in this age group. The latter score was based on 71 evaluations of various tablet intakes in children aged 2 to 4 years regardless of treatment. Table S1 (Supplementary Materials) presents the characteristics of this subset of 71 evaluations from the dataset of 1778 standardized evaluations that gave rise to the reference framework. The barycenter of these evaluations along with the entire confidence ellipses surrounding it, was located in the negatively accepted profile. The difference between acceptability scores was due to important differences between the two subgroups of evaluations for the different observational variables (Table 2).

### 3.3. Acceptability of 500-mg Mebendazole Chewable Tablet According to Age

**Figure 3:**
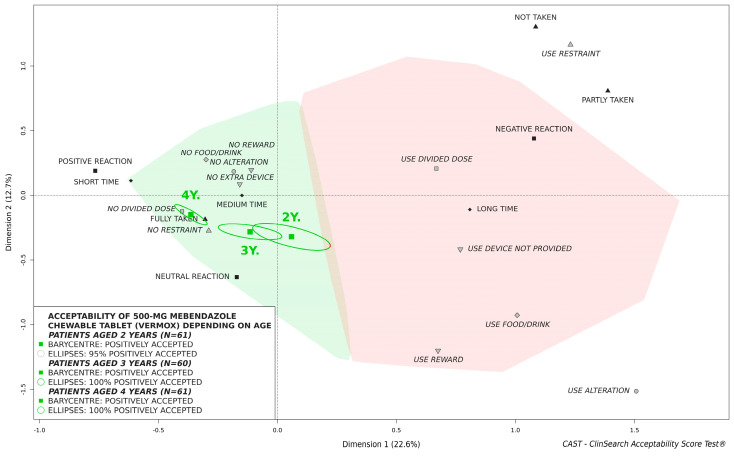
Influence of age on acceptability of 500-mg mebendazole chewable tablet in children aged 2 to 4 years.

**Figure 4:**
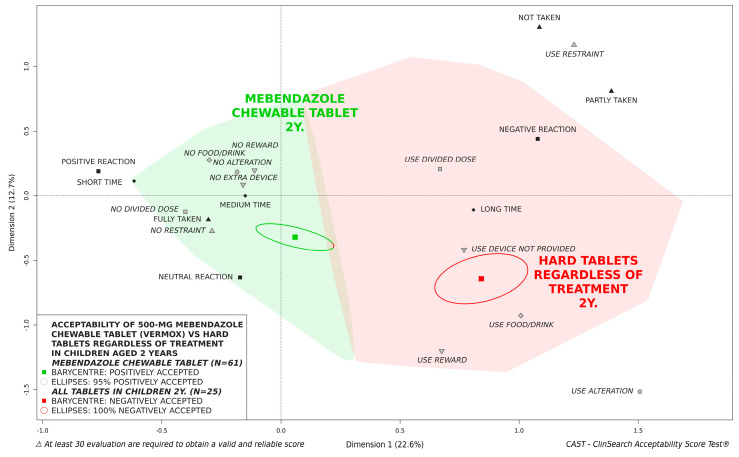
Acceptability of 500-mg mebendazole chewable tablet compared to tablets regardless of treatment in children aged 2 years.

Figure 3 illustrates the acceptability of 500-mg mebendazole chewable tablet in children aged 2, 3, and 4 years. Children aged 4 appeared to be significantly further from the negative area than younger children. The barycenter for children aged 3 were further from the negative area than children aged 2. However, there was no significant difference as confidence ellipses overlapped. A limited part of the confidence ellipses (5%) surrounding the barycenter for children aged 2 years fell in the negatively accepted area. Nevertheless, the chewable tablet tended to be significantly better accepted than hard tablets in those young children (Figure 4).

Acceptability score differences between age groups reflected differences among observer-reported outcomes. Table 3 presents the reported observed measures for each age group describing the many aspects of acceptability. Significant differences were observed for six of the nine observational variables: The patient reaction; the preparation and administration time; dividing the intake of the required dose; using liquid to help with taking the medicine: mainly taking water, and (once) a glass of milk; modifying dosage form prior to administration: dissolving tablet with water; using an extra device: mainly glasses and tongue depressors. Although there was no significant difference for the result of intake, we observed an improvement in acceptability as children grew older.

### 3.4. Influence of Sex and Population Type on Acceptability

**Figure 5:**
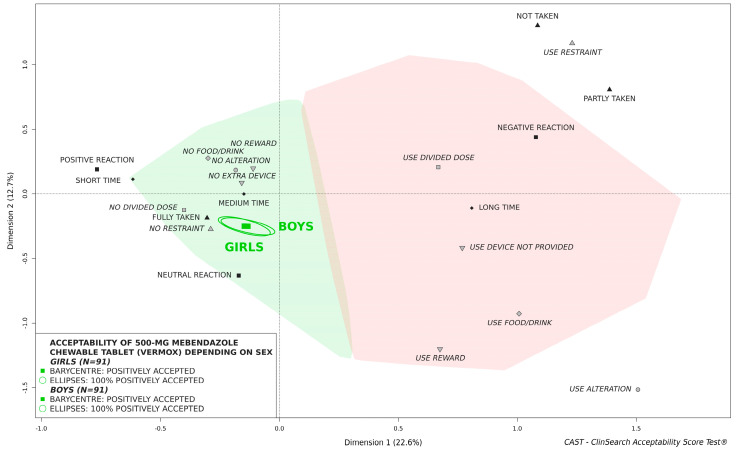
Influence of sex on acceptability of 500-mg mebendazole chewable tablet in children aged 2 to 4 years.

**Figure 6:**
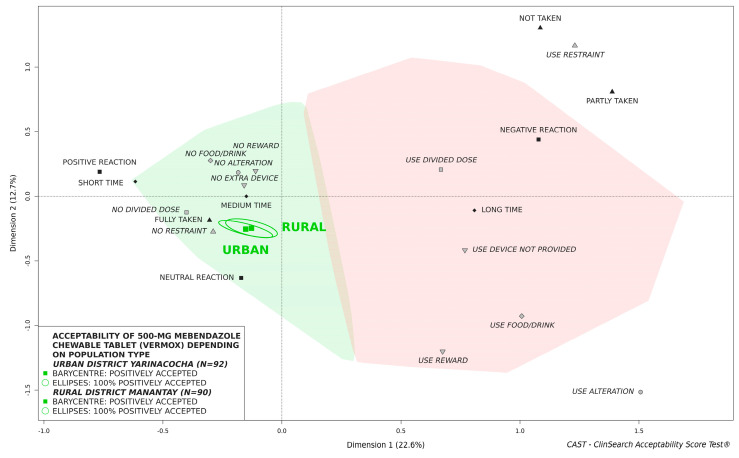
Influence of location on acceptability of 500-mg mebendazole chewable tablet in children aged 2 to 4 years.

The findings indicate that the 500-mg mebendazole chewable tablet was similarly accepted by boys and girls (Figure 5) as well as children from rural and urban district (Figure 6). Stratifying evaluations by age of children, similar findings were observed: in all cases confidence ellipses surrounding the barycenters for boys and girls as well as rural and urban district largely overlapped.

## 4. Discussion

According to the acceptability reference framework, the 500-mg mebendazole chewable tablet was considered as accepted in Peruvian children aged 2 to 4 years as a whole: the medicine was fully located in the green zone of the model defining the “positively accepted” profile. Although only the chewable formulation was administered in children during the deworming campaign, the acceptability reference framework allowed a relative acceptability evaluation: comparing the chewable formulation with conventional tablets regardless of treatment as a surrogate for the original pharmaceutical form of mebendazole. In children aged 2 to 4 years, the chewable formulation of mebendazole was significantly better accepted than hard tablets regardless of treatment. Indeed, the barycenter of the 71 evaluations of tablet intake in this age group from the dataset that gave rise to the acceptability reference framework, and the confidence ellipses surrounding it, were fully located in the red zone of the model defining the “negatively accepted” profile. The chewable formulation improved acceptability significantly for all of the objective measures observed during the medicine intake: the required dose was significantly more fully taken, in a shorter time, with less negative reaction and fewer recourses to methods for achieving administration. Considering the difficulty, or even inability, experienced by young children attempting to swallow SODF [10,14], such difference might be expected as chewable formulation appeared to be a safe and appropriate alternative in pediatrics [20,21,22]. However taste-masking could be challenging for chewable preparations, as the drug is in direct contact with the patient’s taste buds in the mouth and many drugs have a bitter and often aversive taste [23,24]. Adding a combination of excipients (e.g., sweetener, flavor and taste-masking agent) is the simplest method commonly used, sometimes in combination with other taste-masking technologies (e.g., coating, microencapsulation), to improve the palatability of chewable tablets [24,25]. Sweeteners indeed may significantly reduce the bitterness of a drug [26,27]. The 500-mg mebendazole chewable tablet was formulated with a sweetener (sucralose) and flavoring agent (strawberry) reflecting the will to develop a palatable formulation, a key aspect of oral medicine acceptability in children [9]. While improving acceptability in younger children, this SODF retains the advantages of tablets for deworming campaigns in tropical and subtropical areas, e.g., good stability, low risk of incorrect dosing, limited relative cost, easier transport, and storage [10]. In the context of mass drug administration for the control of STH, 500-mg mebendazole administration once or twice a year is recommended to reduce the overall worm burden [28]. Mebendazole formulated as 100-mg/5 mL oral suspension is also on the market. This suspension formulated with sucrose and banana flavor, should be considered to treat STH infections in young children. However, this formulation seems not appropriate for deworming campaigns. Indeed, 25 mL—corresponding to five 5 mL provided dosing cups—would be required to reach the intended required dose of 500-mg per individual. Too large dosing volume is likely to be a barrier to administration in pediatrics [29]. Previous studies indicated that an acceptable volume of liquid is less than 5 mL in toddlers and preschoolers [30,31,32].

Results found that the acceptability increased as children grew older. The 500-mg mebendazole chewable tablet was significantly better accepted in children 4 years of age, while the barycenter for children aged 2 were closer to the negative area. The medicine was positively accepted in children aged 3 and 4 regardless of studied subpopulations. For children aged 2 years of age, a limited part of the confidence ellipses fell in the negative area of the 3D-map. Nevertheless, the chewable formulation was considerably better accepted than hard tablets in children aged 2 to 4 years, even in the younger children aged 2 years, due to an improvement in the different observational variables. According to the summary of product characteristics (SmPC) the chewable formulation should be to chewed completely before swallowing. For younger children, who have difficulty chewing the tablet, an alternate method of administration by first dissolving the tablet in clean water was permitted according to the SmPC. In this study, the alternate method was used for 30% of children aged 2 years, 15% of children aged 3 years, and 5% of children aged 4 years. Although using this permitted alternate method has an impact on the medicine use (e.g., time of preparation, use of methods to achieve administration), drug effectiveness is ensured by the SmPC. For conventional tablets, manipulations are often off-label and may result in critical issues regarding dosing accuracy and bioavailability beyond acceptability issue [33,34,35,36].

There was no influence of sex of children on acceptability. While certain aromas may have contrasting effects on girls and boys, previous study on acceptability of oral antibiotics in children indicated that strawberry flavoring was positively accepted by both girls and boys [17]. In the study of Palmeirim et al., 95% of children reported to have liked the taste of the strawberry flavored chewable tablet [8]. Unpublished results of the first acceptability study conducted in Peru in 2019 indicated that the younger children (in nursery schools), especially from rural areas, were the most distrustful to accept the medicine. Familiar care-givers who spoke to the children in their mother tongue facilitated the medicine intake. In this study there was no influence of population type (urban or rural) on acceptability. Previously published results indicated that healthcare professional skills may positively impact acceptability [17], ensuring for example the intake of the required dose while reducing the preparation and administration time and limiting the use of inappropriate methods to achieve administration in comparison with administration by lay caregivers at home. In this study, medicine was prepared and administered by trained health personnel who regularly give the medicine for deworming campaigns. Furthermore, we should note a particular context of administration due to the COVID-19 situation. Health status is also likely to impact medicine acceptability [9]. In this study, children were not treated for a medical problem, they received chewable mebendazole as a preventive intervention during the deworming campaign.

In this study, we focused on children aged 2 to 4 years. As the 500-mg mebendazole chewable tablets could be used in children aged 12 months and older [7,37], investigating acceptability in subjects up to one year of age should be of interest. Although, chewable formulation of mebendazole is a significant progress for large-scale preventive treatment campaigns in pediatrics, it is necessary to develop new drugs and strategies in preventive chemotherapy for STH to prevent the threat of resistance [38].

## 5. Conclusions

Preventive interventions for STH infections are crucial as those common infections affect deprived communities in tropical/subtropical areas worldwide. Medicine acceptability is at the utmost importance to ensure their effectiveness in pediatrics, reaching a high-risk population. Generating evidence on the acceptability of the new chewable formulation of mebendazole in young children, this study demonstrated that it is an appropriate alternative to the conventional mebendazole hard tablet in preventive chemotherapy for STH in children aged 2 to 4 years.

## Figures and Tables

**Table 1:** Characteristics of the 182 patients included in the study, stratified by patient age.

Characteristics	Patient Age			Statistical Test
	2 Years (*n* = 61)	3 Years (*n* = 60)	4 Years (*n* = 61)	
Sex				χ^2 b^: *p* = 0.98
Female	31 (51) ^a^	30 (50)	30 (49)	
Male	30 (49)	30 (50)	31 (51)	
**District**				χ^2^: *p* = 0.99
Rural (Manantay)	30 (49)	30 (50)	30 (49)	
Urban (Yarinacocha)	31 (51)	30 (50)	31 (51)	
**Place of administration**				χ^2^: *p* = 0.4
Home	38 (62)	41 (68)	45 (74)	
Local facilities	23 (38)	19 (32)	16 (26)	
**Time of administration**				F ^c^: *p* = 0.015
Morning (breakfast)	0 (0)	1 (2)	1 (2)	
Mid-morning	43 (70)	45 (75)	35 (57)	
Noon (lunch)	4 (7)	0 (0)	0 (0)	
Mid-afternoon	14 (23)	14 (23)	25 (41)	
**Treatment exposure**				χ^2^: *p* < 0.001
Previous exposure	4 (7)	28 (47)	25 (41)	
First exposure	57 (93)	32 (53)	36 (59)	

^a^ *n*(%): number and percentages; ^b^ χ^2^: Pearson’s chi-squared test; ^c^ F: Fisher’s exact test.

**Table 2:** Observer-reported outcomes for 500-mg mebendazole chewable tablet compared with tablets regardless of treatment in children aged 2 to 4 years.

Observer-Reported Outcomes	500-mg Mebendazole Chewable Tablet (*n* = 182)	Hard Tablet Regardless of Treatment (*n* = 71)	Statistical Test
**Result intake**			
Fully taken	170 (93) ^a^	49 (69)	F ^b^: *p* < 0.001
Partly taken	11 (6)	22 (31)	
Not taken	1 (1)	0 (0)	
**Patient reaction**			
Positive	65 (36)	13 (18)	χ^2 c^: *p* < 0.001
Neutral	91 (50)	22 (31)	
Negative	26 (14)	36 (51)	
**Preparation and ** **administration time**			
Short	22 (12)	9 (13)	χ^2^: *p* < 0.001
Medium	131 (72)	24 (34)	
Long	29 (16)	38 (54)	
**Divided dose**			
No divided dose	163 (90)	30 (42)	χ^2^: *p* < 0.001
Use divided dose	19 (10)	41 (58)	
**Food/drink** ^d^			
No food/drink	95 (52)	18 (25)	χ^2^: *p* < 0.001
Use food/drink	87 (48)	53 (75)	
**Alteration** ^e^			
No alteration	152 (84)	15 (21)	χ^2^: *p* < 0.001
Use alteration	30 (16)	56 (79)	
**Extra device** ^f^			
No extra device	152 (84)	55 (77)	χ^2^: *p* = 0.35
Use extra device	30 (16)	16 (23)	
**Reward**			
No reward	174 (96)	36 (51)	χ^2^: *p* < 0.001
Use reward	8 (4)	35 (49)	
**Restraint**			
No restraint	182 (100)	59 (83)	χ^2^: *p* < 0.001
Use restraint	0 (0)	12 (17)	

^a^ *n* (%): number and percentages; ^b^ F: Fisher’s exact test; ^c^ χ^2^: Pearson’s chi squared test; ^d^ either mixed with the drug or taken just before or after administration; ^e^ modification of dosage form prior to administration; ^f^ device not provided with the medicine.

**Table 3:** Observer-reported outcomes, stratified by patient age.

Observer-Reported Outcomes	Patient Age			Statistical Test
	2 Years (*n* = 61)	3 Years (*n* = 60)	4 Years (*n* = 61)	
**Result intake**				
Fully taken	55 (90) ^a^	55 (92)	60 (98)	F ^b^: *p* = 0.21
Partly taken	5 (8)	5 (8)	1 (2)	
Not taken	1 (2)	0 (0)	0 (0)	
**Patient reaction**				
Positive	7 (11)	27 (45)	31 (51)	χ^2 c^: *p* < 0.001
Neutral	36 (59)	26 (43)	29 (48)	
Negative	18 (30)	7 (12)	1 (2)	
**Preparation and administration time**				
Short	3 (5)	5 (8)	14 (23)	χ^2^: *p* < 0.001
Medium	36 (59)	48 (80)	47 (77)	
Long	22 (36)	7 (12)	0 (0)	
**Divided dose**				
No divided dose	50 (82)	53 (88)	60 (98)	χ^2^: *p* = 0.012
Use divided dose	11 (18)	7 (12)	1 (2)	
**Food/drink** ^d^				
No food/drink	35 (57)	21 (35)	39 (64)	χ^2^: *p* = 0.004
Use food/drink	26 (43)	39 (65)	22 (36)	
**Alteration** ^e^				
No alteration	43 (70)	51 (85)	58 (95)	χ^2^: *p* = 0.001
Use alteration	18 (30)	9 (15)	3 (5)	
**Extra device** ^f^				
No extra device	43 (70)	51 (85)	58 (95)	χ^2^: *p* = 0.001
Use extra device	18 (30)	9 (15)	3 (5)	
**Reward**				
No reward	57 (93)	57 (95)	60 (98)	F: *p* = 0.44
Use reward	4 (7)	3 (5)	1 (2)	
**Restraint**				
No restraint	61 (100)	60 (100)	61 (100)	
Use restraint	0 (0)	0 (0)	0 (0)	

^a^ *n* (%): number and percentages; ^b^ F: Fisher’s exact test; ^c^ χ^2^: Pearson’s chi squared test; ^d^ either mixed with the drug or taken just before or after administration; ^e^ modification of dosage form prior to administration; ^f^ device not provided with the medicine.

## Data Availability

Data underlying the study cannot be made publicly available due to legal and ethical considerations. European Union (GDPR) and French (Law n° 78–17 of 6 January 1978) laws restrict the public sharing of personally identifiable data. Requests for data will be processed according to the French MR-003 Code of conduct by the data controller, ClinSearch, which allows for the use of data for the purpose of reproducing study results. Requests to access the data for this purpose may be sent to the data protection officer of ClinSearch: dataprivacy@clinsearch.net and researchers outside the European Union will need to sign a transfer agreement.

## Supplementary Materials

The following are available online at www.mdpi.com/article/10.3390/pharmaceutics14010027/s1. Table S1: Patient and medicine characteristics of the 71 evaluations of tablet intake in children from 2 to 4 years of age within the dataset that gave rise to the acceptability reference framework.
