# Supplementary material for: Acceptability of Mebendazole Chewable Tablet in Children Aged 2 to 4 Years in Peru
Source: Pharmaceutics. 2021 Dec 23;14(1):27. doi: 10.3390/pharmaceutics14010027 (PMC8780656; doi:10.3390/pharmaceutics14010027)
Supplement: Supplementary file 1 [file pharmaceutics-14-00027-s001.zip › pharmaceutics-1488325-supplementary.pdf]

pharmaceutics

Supplementary Materials: Acceptability of Mebendazole Chewable Tablet in Children Aged 2 to 4 Years in Peru

Fernando Perez, Thibault Vallet, Zarela Bravo, Kristin Callahan and Fabrice Ruiz

Table S1. Patient and medicine characteristics of the 71 evaluations of tablet intake in children from 2 to 4 years of age within the dataset that gave rise to the acceptability reference framework.

Characteristics	*n* (%)
**Sex**	
Female	34 (48)
Male	37 (52)
**Age**	
2 years	25 (35)
3 years	23 (32.5)
4 years	23 (32.5)
**Treatment exposure**	
Previous exposure	35 (49)
First exposure	36 (51)
**Setting**	
Community	6 (8)
Hospital	65 (92)
**Country**	
Morocco	39 (55)
India	25 (35)
France	4(6)
Norway	3 (4)
**2nd level (therapeutic subgroup) of the Anatomical Therapeutic Chemical (ATC) classification system**	
Antiepileptics	14 (20)
Psycholeptics	9 (13)
Antivirals	9 (13)
Antibacterials	9 (13)
Antithrombotic agents	8 (11)
Corticosteroids	4 (5)
Other (<5%)	18 (25)
